# CrSbSe_3_ Nanowire: A Platform for Near-Perfect Spin Selectivity

**DOI:** 10.1021/acs.nanolett.6c02702

**Published:** 2026-08-21

**Authors:** Alyssa Horne, Yongmei M. Jin, Ranjit Pati

**Affiliations:** † Department of Physics, 3968 Michigan Technological University, Houghton, Michigan 49931, United States; ‡ Department of Materials Science and Engineering, 3968 Michigan Technological University, Houghton, Michigan 49931, United States; § Henes Center for Quantum Phenomena, 3968 Michigan Technological University, Houghton, Michigan 49931, United States

**Keywords:** ferromagnetism, spin filter, semiconducting nanowire, density functional theory, quantum transport

## Abstract

Spin-selective carrier transport in low-dimensional magnetic semiconductors is central to the development of next-generation spintronics. However, achieving robust spin-filtering together with long spin-coherence under nonequilibrium conditions remains a major challenge. Recently isolated by top-down exfoliation, the CrSbSe_3_ nanowire has emerged as a ferromagnetic semiconductor exhibiting enhanced coercivity compared to its bulk counterpart. Here, using first-principles density functional theory combined with quantum transport calculations, we demonstrate that the CrSbSe_3_ nanowire is a ferromagnetic semiconductor with a band gap of 1.26 eV. We identify superexchange interactions mediated by Se­(p-orbitals) as the primary mechanism stabilizing its ferromagnetic order. Noncollinear spin calculations including spin–orbit coupling reveal an unchanged band topology near the Fermi level, suggesting long spin-coherence. Quantum transport calculations reveal robust spin-selective conduction, with the spin-injection factor approaching 100% for biases up to 0.6 V, independent of channel length, establishing the CrSbSe_3_ nanowire as a promising spin-filtering platform for spintronics.

## Graphical abstract

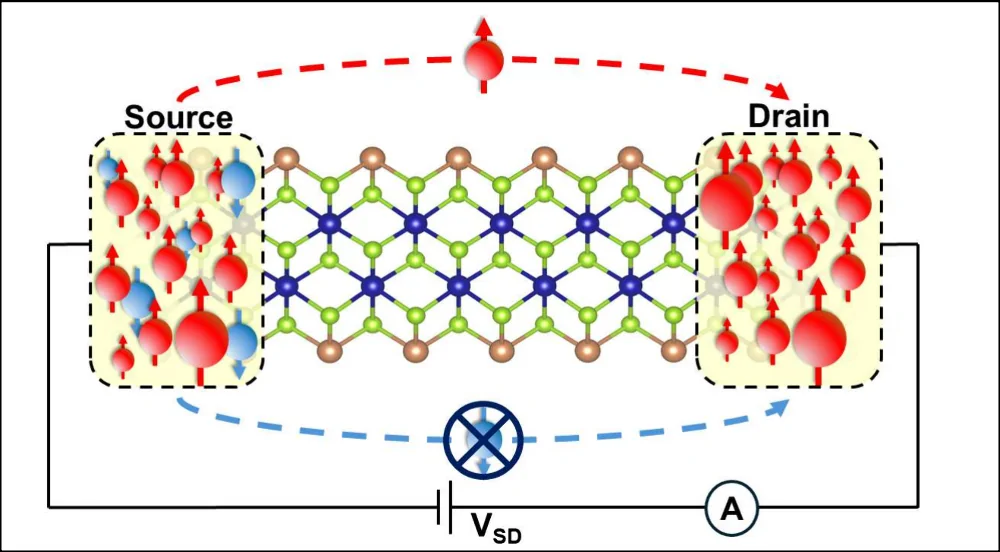

Spintronics, which exploits the electron’s spin for information storage, processing and offers a transformative pathway beyond the scaling limits of conventional charge-based electronics.^1–7^ Harnessing the electron spin degree of freedom in a low-dimensional semiconductor is therefore critical for advancing next-generation spintronics.^1–12^ This would enable ultracompact, energy efficient devices that integrate memory and logic on a single platform.^1–10^

Spin functionality can be incorporated into semiconductors via two principal approaches: the injection of spin-polarized carriers from a ferromagnetic metal electrode into a semiconducting channel,^4,10,13–16^ or the realization of spin-polarized electronic states within the semiconductor itself.^17–23^ Despite its conceptual simplicity, the former strategy faces fundamental limitations. In particular, the pronounced conductivity mismatch between ferromagnetic metals and semiconductors severely limits spin injection efficiency by causing substantial spin depolarization at the interface.^24^ Furthermore, the lack of available electronic states near the Fermi level suppresses efficient spin transfer to semiconductor. To overcome the conductivity mismatch, insulating tunnel barriers are commonly introduced between the ferromagnetic metal electrode and the semiconductor,^25–29^ enabling more efficient spin injection. The tunnel-barrier concept has recently been extended to antiferromagnetic and altermagnetic tunnel junctions.^30–32^ Despite these significant advances, all of these architectures rely on spin injection from magnetic metal electrodes. In contrast, semiconductors with intrinsically spin-polarized electronic states provide a more direct and potentially robust route toward realizing spin functionality.

Initial efforts to realize low-dimensional semiconductors with intrinsic spin-polarized states were met with significant skepticism due to the Mermin-Wagner theorem,^33^ which predicted thermal fluctuations to cause spin depolarization in low-dimensional isotropic units. However, the realization of stable long-range magnetic ordering in two-dimensional (2D) van der Waals (vdW) crystals down to monolayer limit at a finite temperature, as demonstrated in materials such as CrI_3_,^34^ Cr_2_Ge_2_Te_6_,^35^ and Fe_3_GeTe_2_,^36,37^ has ignited research interest in low-dimensional spintronics. For example, layered CrI_3_ has been used as a tunnel barrier in magnetic tunnel junction devices that exhibit excellent spin filtering and giant tunnel magnetoresistance as high as 57000%.^38–41^

Extending this concept, a stable one-dimensional (1D) semiconducting nanowire with an intrinsic spin polarized state would offer more compact device integration and further advance scalable spintronics architectures. The recent successful exfoliation of CrSbSe_3_ nanowire from its quasi-one-dimensional vdW crystal counterpart^42–46^ using a top-down approach^47^ that is less prone to defect formation provides a novel platform for spin selectivity in the 1D regime. This nanowire is reported to exhibit robust long-range ferromagnetic ordering^47–49^ with enhanced coercivity (by as much as 1 order of magnitude) and remanence than its bulk counterpart.^47^ In addition, they are found to remain air stable with no magnetic degradation for an extended period of time^49^ and also have been predicted to possess a Curie temperature of ∼ 170 K.^48^ The presence of ferromagnetic ordering and semiconducting behavior has also been confirmed in this nanowire synthesized using a template assisted bottom-up approach.^49^ Hence, the natural question to ask is can such nanowires function as efficient spin filtering tunnel barriers under nonequilibrium conditions — *an important prerequisite for its usage in next-generation data storage and sensing technologies*? We use density functional theory (DFT) in conjunction with a single-particle Green’s function approach to address this question.

Using a finite segment of CrSbSe_3_ nanowire as the scattering channel in an open device model, we probe its spin filtering capability through spin-resolved quantum transport calculations. Our calculations show a pronounced imbalance between majority- and minority-spin currents. Orbital analysis reveals that this spin-selective transport originates from the distinct orbital character of the conducting states. Specifically, the majority-spin transport is dominated by a Cr­(d)–Se­(p)–Cr­(d) hybridized network spanning the scattering channel, which provides highly efficient conducting pathways. In contrast, minority-spin states are nearly absent within the bias-induced chemical potential window. The highest occupied minority-spin state that enters the chemical potential window only at higher bias is derived almost exclusively from Se-p orbitals, with negligible contribution from Cr-d states, resulting in much weaker electronic coupling and a substantially lower minority-spin current. Consequently, the spin injection factor (SIF) reaches 100% for biases up to ∼0.6 V and remains essentially insensitive to channel length, highlighting the robustness of spin-selectivity and establishing the CrSbSe_3_ nanowire as a promising one-dimensional platform for perfect spin filtering.

CrSbSe_3_ is a van der Waals crystal with quasi-one-dimensional character, formed from weakly bound CrSbSe_3_ nanowires.^50^ We therefore adopted the nanowire motif extracted from the bulk crystal as the initial structure for our atomic model, and performed full structural relaxation without symmetry constraints using spin-polarized density functional theory (DFT) within periodic boundary conditions^51^ to obtain the equilibrium geometry and electronic structure. All calculations were carried out using the Vienna Ab initio Simulation Package (VASP) within a plane wave basis, employing the projector augmented-wave (PAW) method and the Perdew–Burke–Ernzerhof (PBE) generalized gradient approximation (GGA) for the exchange–correlation functional.^52–56^ The electronic band structure was additionally calculated using the HSE06 hybrid functional and noncollinear spin calculations including spin–orbit coupling (SOC).^57–59^ To investigate the spin-dependent quantum transport properties of the CrSbSe_3_ nanowire, we resorted to a real space, spin-unrestricted, orbital-dependent *a posteriori* DFT formalism. The real space approach enables partitioning of the Kohn–Sham–Fock matrix to construct the bias-dependent Green’s function^60–62^ and calculate the spin-polarized current within a spin-conserved tunneling framework.^23,62^ Atom-centered Gaussian basis functions^63^ and the Becke three-parameter hybrid functional (B3LYP)^63,64^ were used, which have been shown to provide improved descriptions of the electronic structure of semiconductors and quantum transport properties in nanoscale junctions.^65^ It is worth noting that the density of states obtained using B3LYP for 3d transition-metal systems has been reported to be in good agreement with results from embedded dynamical mean-field theory, providing further support for the reliability of using B3LYP to calculate spin-dependent transport.^66^ Detailed descriptions of the device model and computational methodology are provided in the Supporting Information.

Figure 1 illustrates the optimized ground-state structure of the CrSbSe_3_ nanowire from both cross-sectional (Figure 1a) and longitudinal (Figure 1b) perspectives. The equilibrium lattice constant was determined to be 3.75 Å, which agrees well with a previous study.^48^ The relaxed free-standing nanowire retains structural parameters very similar to those of the corresponding one-dimensional units in the parent van der Waals crystal,^50^ indicating minimal structural reconstruction of the nanowire upon exfoliation. To determine the magnetic ground state, the total energies of the ferromagnetic (FM) and antiferromagnetic (AFM) configurations were evaluated as a function of lattice parameter, as shown in Figure 1(c). The FM configuration is energetically favored over the AFM state, with an exchange energy, *E*_XC_ = *E*_FM_ – *E*_AFM_, of −47.47 meV, demonstrating robust ferromagnetic coupling between neighboring Cr atoms. The calculated magnetic moment on each Cr atom is approximately 3.1 μ_B_, consistent with neutron diffraction derived magnetic moment of 3.06 μ_B_/Cr,^47^ while the nearest-neighbor Se atoms acquire a small induced magnetic moment of about −0.16 μ_B_, as shown in the inset in Figure 1c. The observed antiparallel spin polarization on Se strongly suggests that the ferromagnetic ordering originates from indirect exchange interactions mediated through Cr­(d)–Se­(p)–Cr­(d) hybridization as also observed in our earlier work.^50^ Such superexchange-driven coupling stabilizes the FM ground state and suppresses the antiferromagnetic tendency typically associated with elemental Cr-based systems.^23^ Furthermore, the magnetic anisotropy energy is calculated to be 0.13 meV/Cr, with the easy magnetization axis oriented perpendicular to the wire axis. The presence of finite out-of-wire axis magnetic anisotropy is particularly significant for a low-dimensional magnetic nanowire, as it helps stabilize long-range ferromagnetic ordering against thermal spin fluctuations, which is also evident from the experimental observation of strong magnetic hysteresis and enhanced coercivity in the free-standing nanowire compared to its bulk counterpart.^47^

**Figure 1:**
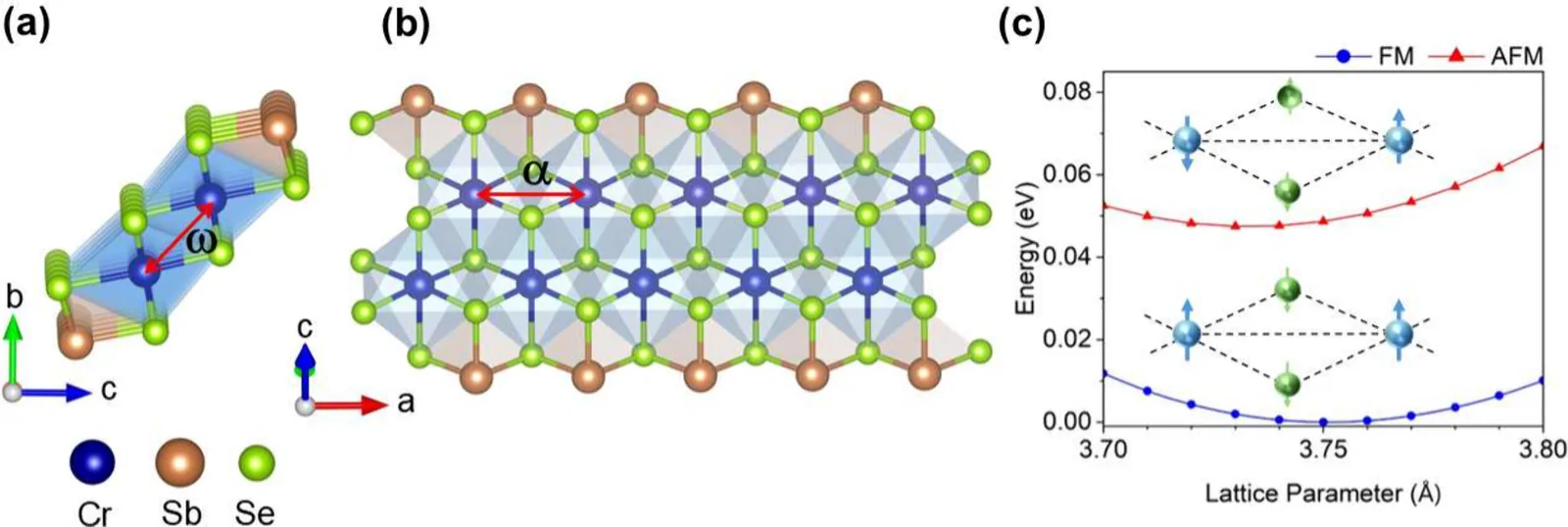
Optimized ground state structure of CrSbSe_3_ nanowire. (a) Cross-sectional and (b) longitudinal view of the magnetic nanowire. The first and second nearest Cr–Cr distances are represented by ω and α, respectively, where ω = 3.57 Å, and α = 3.75 Å. The energy for the ferromagnetic (FM) and antiferromagnetic (AFM) states as a function of lattice parameter, with respect to the ground state (FM) energy of the system, are shown in panel (c). The inset portrays the spin alignment for the respective configurations. The FM state is energetically stable by ∼0.05 eV as compared to the AFM configuration.

Figure 2 compares the spin-polarized electronic band structures of the CrSbSe_3_ nanowire obtained using the generalized gradient approximation within the PBE functional, the noncollinear PBE approach including spin–orbit coupling (SOC) and the hybrid HSE06 functional, enabling a systematic evaluation of exchange-correlation and relativistic corrections on the electronic properties. The PBE functional reveals (Figure 2a) the CrSbSe_3_ nanowire as an indirect-gap semiconductor with a band gap of 0.35 eV. The valence band maximum (VBM) occurs closer to the Γ-point, whereas the conduction band minimum (CBM) lies closer to the X-point, indicating momentum-space separation between the occupied and unoccupied states. Such an indirect-gap behavior is intrinsic to low-dimensional systems where anisotropic orbital overlap and directional bonding dominate the electronic dispersion. Importantly, the strong separation between the majority-spin (MJ) and minority-spin (MN) bands demonstrates pronounced exchange splitting arising from the localized Cr 3d magnetic moments. The presence of several avoided crossings in the MJ bands along the Γ–X path is also noticeable from Figure 2a (see Figure S1 in Supporting Information). These anticrossing features indicate strong hybridization between bands possessing compatible symmetry and orbital character. Orbital-resolved band structure analysis (see Figure S1) reveals that these avoided crossings are accompanied by orbital character switching between the Cr d orbitals. For example, the MJ band originating near −0.52 eV at the Γ-point evolves from predominantly *d*_*yz*_ character into *d*_*xz*_ character after the avoided crossing region between Γ and X. A similar orbital interchange is observed for the MJ band originating near −0.86 eV at the Γ-point. Importantly, the evolution of orbital character across reciprocal space (see Figure S1) indicates strong hybridization between Cr d-orbitals and neighboring Se p-states, providing the microscopic basis for the superexchange-mediated stabilization of ferromagnetic ordering. Particularly, the symmetry and orbital composition of these electronic states have direct implications for how they respond to an applied bias and consequently govern the spin-dependent transmission characteristics of the nanowire. The calculated PBE band gap of 0.35 eV is close to the previously reported value of 0.32 eV for the bulk van der Waals CrSbSe_3_ crystal obtained using the same exchange–correlation functional.^50^ This observation suggests that the van der Waals coupling between neighboring nanowire units in the crystal contributes minimally to the frontier electronic states, and the free-standing nanowire preserves the essential electronic characteristics of the parent crystal despite dimensional confinement.

**Figure 2:**
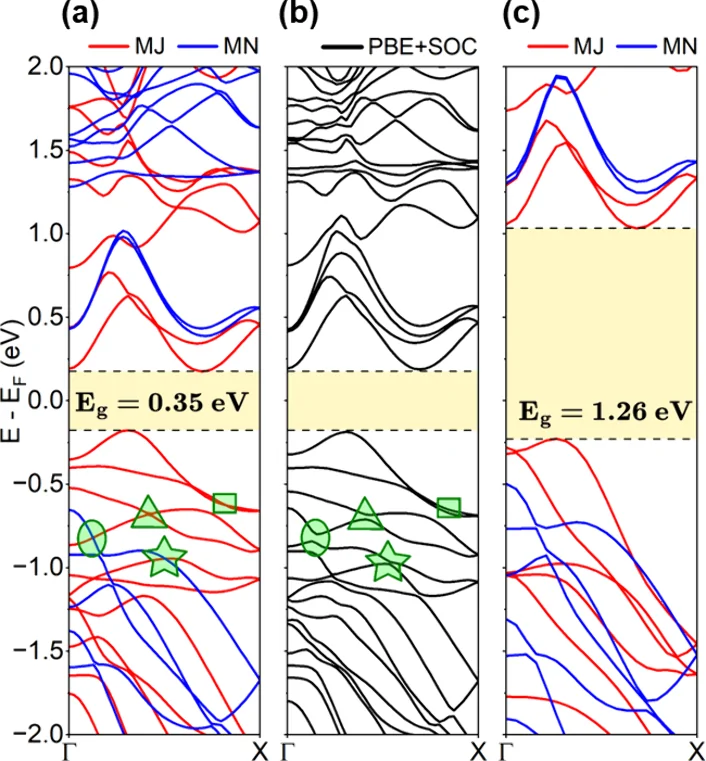
Spin-polarized electronic band structures along the high-symmetry path Γ–X in the Brillouin zone. Results obtained using the (a) PBE functional, (b) PBE that includes spin–orbit coupling (SOC), and (c) the HSE06 hybrid functional. Majority (MJ) and minority (MN) spin channels are represented by red and blue, respectively, with an energy band gap (*E_g_*) highlighted in yellow. The shaded symbols in (a) show band degeneracy. The corresponding SOC-induced band splittings are shown in (b). The magnitude of the respective band splittings are 5.18 meV (square), 25.51 meV (triangle), 40.44 meV (circle) and 14.85 meV (star).

The influence of relativistic effects was investigated through noncollinear spin calculations including SOC, as shown in Figure 2(b). The overall band topology and indirect gap nature remain largely unchanged after SOC inclusion, indicating that the electronic structure is primarily governed by exchange interactions and crystal-field splitting rather than strong relativistic effects. However, SOC lifts the band degeneracies present at several k-points in Figure 2(a), producing distinct band splittings highlighted by the corresponding shaded symbols in Figure 2(b). Importantly, these SOC-induced splittings occur at energies well below the Fermi level, with the most pronounced splitting located approximately 0.6 eV below the Fermi energy. Consequently, these states lie outside the bias-induced chemical potential window considered in the present study (up to approximately 0.5 eV below and above the Fermi energy for an applied bias of 1 V) and therefore do not contribute appreciably to the spin-polarized current. Moreover, the SOC-induced splittings, which are on the order of a few tens of meV, are comparable to the energy broadening of the transport states arising from their coupling to the semi-infinite electrodes. As a result, these splittings are effectively smeared in the transmission spectrum. These observations thus support the use of the spin-conserved tunneling formalism for the bias range investigated in this work.

To more accurately describe the electronic structure of the quasi-particle and mitigate the self-interaction error inherent in semilocal functionals, the HSE06 hybrid functional was employed. As shown in Figure 2c, inclusion of screened exact exchange leads to a substantial widening of the band gap from 0.35 to 1.26 eV (only the unoccupied bands shift upward). Despite this quantitative correction, the indirect nature of the band gap remains unchanged, demonstrating that the fundamental band topology predicted by PBE is robust against exchange-correlation refinement. The pronounced increase in the band gap is consistent with the known tendency of semilocal density functionals to underestimate the electronic band gap due to overdelocalization of Cr d-states.^23^ The larger HSE06 band gap has several important implications. First, it indicates enhanced electronic localization and stronger exchange interactions for Cr-derived states. Second, the corrected gap places the material within an energetically favorable range for the optoelectronic applications.

Motivated by the pronounced spin polarization between the majority- and minority-spin bands, the weak spin–orbit interaction near the Fermi level, and the semiconducting nature of the nanowire, we constructed a two-terminal device shown in Figure 3(a) to investigate its spin-dependent transport behavior under nonequilibrium conditions and evaluate its potential as an efficient spin-filtering channel for spintronic applications. An external bias (*V*_SD_) was applied to induce nonequilibrium current through the nanowire. The bias effect was included explicitly within the Green’s function formalism,^23,62^ thereby accounting for the bias-induced redistribution of charge density and electrostatic potential across the junction. The evolution of the planar-averaged electrostatic potential^67^ under different applied biases is shown in Figure 3(b). Upon application of *V*_SD_, the electrochemical potentials of the source and drain electrodes shift in opposite directions, producing a nonequilibrium carrier imbalance between the two contacts.^60,61^ This redistribution results in charge accumulation at one electrode and charge depletion at the other, giving rise to a finite electric field across the scattering region. The electrostatic potential profiles exhibit an approximately linear potential drop across the nanowire channel, while the electrode regions maintain nearly constant potentials characteristic of metallic reservoirs in local equilibrium.

**Figure 3:**
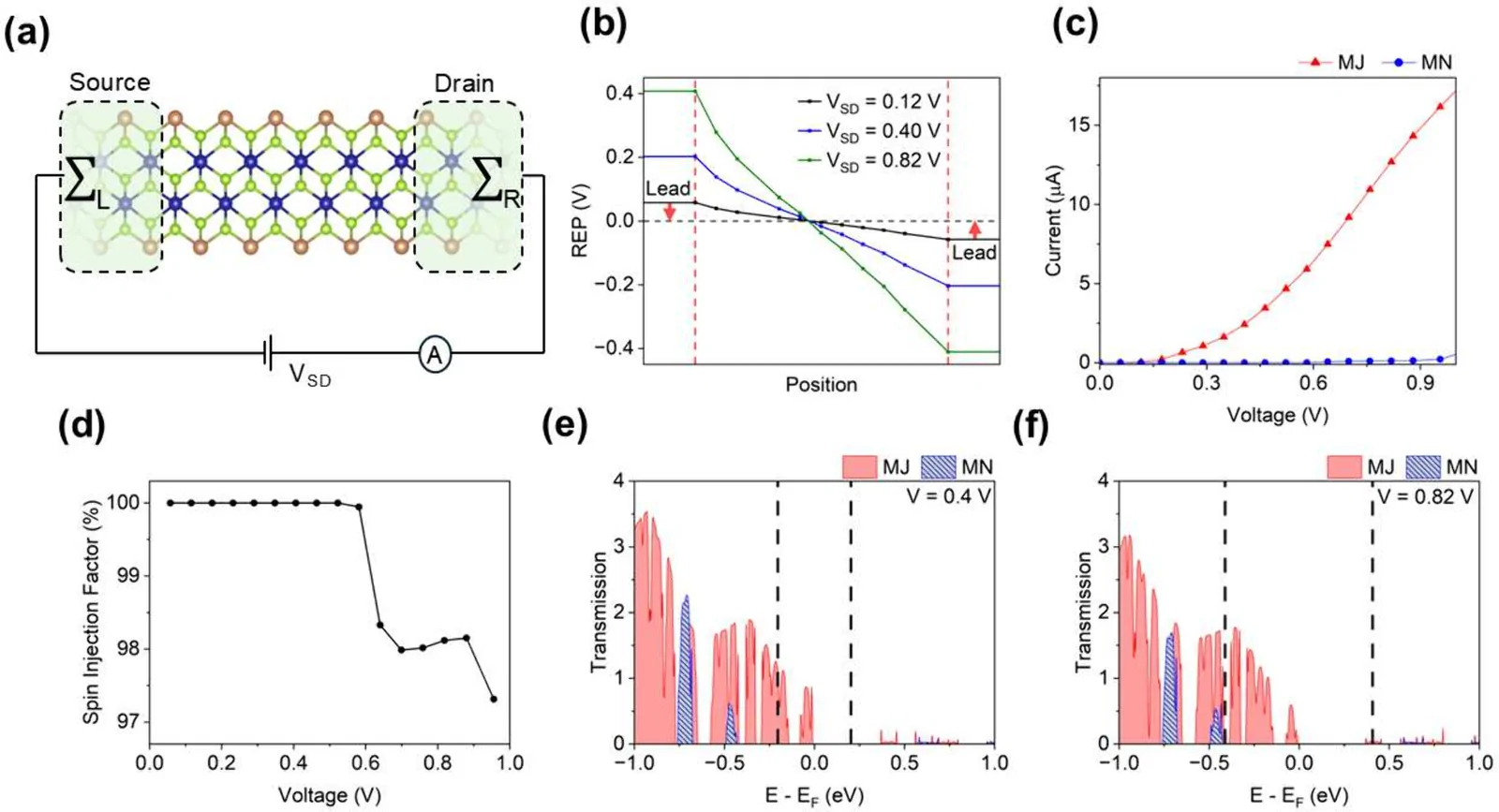
Spin-dependent transport properties of the CrSbSe_3_ nanowire. (a) Open device configuration for a finite CrSbSe_3_ nanowire channel coupled to semi-infinite source (∑_L_) and drain (∑_R_) electrodes. The applied bias voltage is labeled by *V*_SD_ and the resulting current by *A*. (b) Bias dependent planar average electrostatic potential (REP) profile at different atomic positions along the wire axis in the nanowire junction. The dotted horizontal line at REP = 0.0 V (Fermi level) denotes the equilibrium condition, where the source and drain are at the same electrochemical potential. (c) The calculated spin-dependent current as a function of applied bias; MJ refers to majority spin state and MN to minority spin state. (d) Spin-injection factor vs applied bias; it remains above 97% up to ∼1 V. Spin-resolved transmission function at applied bias of (e) 0.40 V and (f) 0.82 V. The dashed vertical lines show the respective chemical potential windows.

The calculated spin-resolved current as a function of applied bias is presented in Figure 3(c). A robust spin-selective transport behavior emerges beyond approximately 0.1 V, where the MJ channel enters an ON state characterized by a rapidly increasing current, whereas the MN channel remains effectively blocked with nearly zero current. This behavior demonstrates a strong bias-induced spin filtering effect. Even at higher biases, the minority spin current remains several orders of magnitude smaller than the majority spin contribution. Although a finite MN current begins to appear beyond approximately 0.6 V, its magnitude remains negligible compared to the MJ current. The observed asymmetry in the spin-resolved current can be understood from the asymmetric spin-resolved electronic structure near the Fermi level. As shown in Figure 2, for both the PBE and HSE06 calculations, the occupied majority-spin bands lie substantially closer to the Fermi level than the occupied minority-spin bands. Under an applied bias, charge transport is primarily governed by the electronic states that fall within the bias-induced chemical potential window around the Fermi level (Figure 3b). Consequently, majority-spin states are brought into this chemical potential window even at relatively low bias, providing available conducting channels and producing a measurable majority-spin current. In contrast, the minority-spin states remain farther from the Fermi level and therefore lie outside the chemical potential window, suppressing their contribution to the current until a sufficiently large bias is applied.

To quantify the efficiency of spin-selective carrier injection, we calculated the spin-injection factor (SIF),^68^ $\eta =\frac{I_{\mathrm{MJ}}-I_{\mathrm{MN}}}{I_{\mathrm{MJ}}+I_{\mathrm{MN}}}\times 100\%$ , defined in terms of the relative contributions of the majority- and minority-spin currents. As shown in Figure 3(d), the SIF remains essentially 100% up to approximately 0.6 V, confirming nearly perfect spin-polarized transport through the nanowire junction. Beyond this bias, the gradual emergence of minority-spin transmission channels leads to a slight reduction in η; nevertheless, the SIF remains exceptionally high, exceeding approximately 97–98% up to ∼1 V. Such robust spin polarization under finite bias highlights the remarkable efficiency of spin injection from the electrodes into the nanowire channel and demonstrates the capability of the CrSbSe_3_ nanowire to sustain highly spin-polarized currents under realistic conditions.

To understand the microscopic origin of the bias-dependent spin transport, we analyzed the spin-resolved transmission spectra at representative biases of 0.40 and 0.82 V, shown in Figure 3(e) and (f), respectively. The transmission function represents the total transmission probability summed over all available conducting channels and directly determines the magnitude of the current within the chemical-potential window defined by the applied bias. At 0.40 V, the majority spin transmission exhibits substantial spectral weight within the bias window, whereas the minority spin transmission is nearly absent. Consequently, only majority-spin carriers contribute to electrical conduction, explaining both the large MJ current and the nearly perfect spin injection efficiency observed at low bias. The complete suppression of minority-spin transmission within the transport window demonstrates the half-metallic-like transport behavior of the nanowire junction under moderate bias conditions. At the higher bias of 0.82 V, the finite number of minority-spin transmission states originating from the occupied states below the Fermi level gradually starts to enter the chemical potential window. These newly accessible MN transmission channels give rise to a nonzero minority spin current and account for the gradual reduction in the spin injection factor beyond 0.6 V. Simultaneously, the majority-spin transmission near the Fermi level decreases in intensity compared with that in the lower-bias case, further contributing to the reduction in spin polarization. Nevertheless, the majority-spin contribution remains dominant throughout the entire bias window, thereby preserving the strongly spin-polarized nature of the total current. To gain deeper orbital-level insight into the observed asymmetry in the spin-dependent transmission, we analyzed the highest occupied molecular orbitals (HOMOs) of the majority- and minority-spin channels in the scattering region of the device (Figure S2). The majority-spin HOMO is composed of hybridized Cr d and Se p orbitals, providing further confirmation of the role of Se (p) mediated Cr d-Se p hybridization in the superexchange interaction that stabilizes the long-range ferromagnetic ordering. In contrast, the minority-spin HOMO is derived almost exclusively from Se p orbitals, with negligible Cr contribution. This distinct difference in orbital character suggests that the Cr­(d)–Se­(p)–Cr­(d) hybridized network forms the dominant transport pathway for majority-spin electrons, thereby accounting for the substantially higher majority-spin current.

Figure 4 demonstrates the effect of channel length on spin-dependent transport. As expected for a semiconducting tunnel barrier, both majority- and minority-spin currents in CrSbSe_3_ nanowire decrease with increasing channel length, with the shortest nanowire channel exhibiting a higher current than the longest channel. The majority-spin current (Figure 4a) begins to increase beyond an applied bias of ∼0.1 V, whereas the minority-spin current (Figure 4b) remains nearly zero up to 0.6 V, resulting in a spin-injection factor (Figure 4c) close to 100% for a bias of up to 0.6 V for all channel lengths. The near-zero minority-spin current can be attributed to the absence of available minority-spin channels within the chemical potential window, as shown in Figure 3e. At higher bias, however, the spin-resolved currents exhibit a noticeable nonlinear behavior. This arises because the majority- and minority-spin transport channels possess distinct orbital characters (see Figure S2) and therefore respond differently to the applied electric field. As the bias increases, the spin-resolved eigenchannel energies evolve nonlinearly due to higher-order Stark effects,^68^ producing unequal energy shifts and orbital rehybridization of the transport channels. These field-induced modifications alter the energy alignment and coupling of the transport channels to the lead within the bias window, leading to the observed nonlinear evolution of the spin-polarized current. The longer channel (higher tunnel barrier height) maintains a high spin-injection factor even beyond 0.6 V due to the stronger suppression of minority-spin conduction at higher bias (Figure 4b). These results indicate a trade-off between current magnitude and spin selectivity, where shorter channels favor higher current, while longer channels provide improved spin-filtering performance.

**Figure 4:**
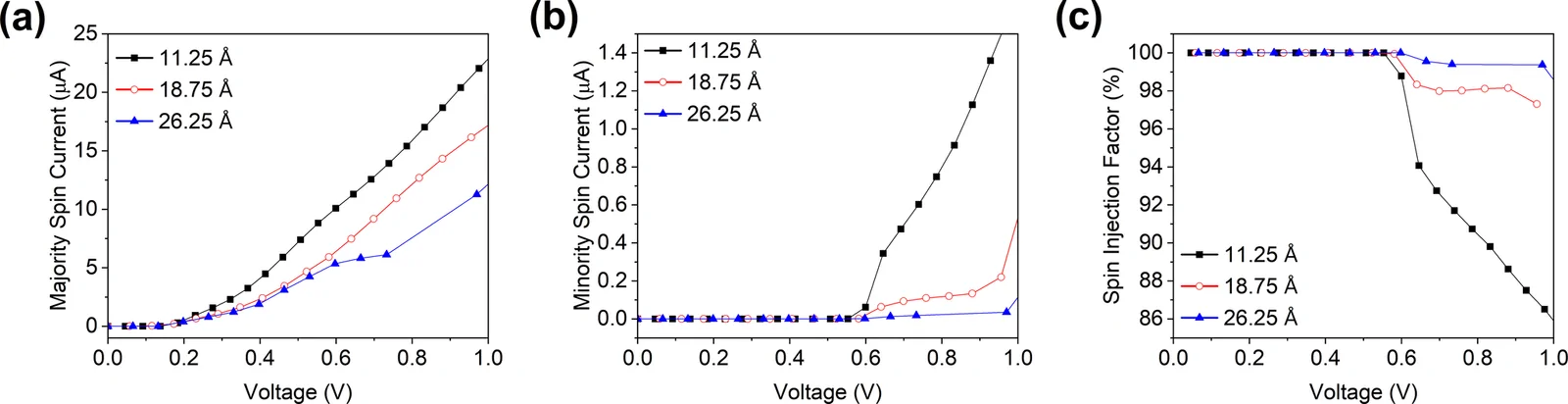
Dependence of spin-selectivity on channel length. Bias-dependent (a) majority spin current and (b) minority spin current as a function of channel length. (c) The spin-injection factor for different channel lengths.

In summary, we present a comprehensive investigation of the spin-selective properties of the CrSbSe_3_ nanowire. Using spin-unrestricted density functional theory, we demonstrate that the CrSbSe_3_ nanowire is a ferromagnetic semiconductor with an indirect band gap of 1.26 eV. The stable ferromagnetic ordering, characterized by an exchange energy of −47.47 meV, originates from a superexchange mechanism mediated by Se p-states. Relativistic effects examined through noncollinear spin calculations, including spin–orbit coupling, are found to have a negligible influence on the band topology and the indirect-gap nature of the system, indicating that the electronic structure near the Fermi level is predominantly governed by exchange interactions and crystal-field splitting. Quantum transport calculations incorporating finite-bias effects explicitly reveal highly spin-selective conduction, with the spin injection factor reaching nearly 100% for biases up to 0.6 V, independent of channel length. Furthermore, increasing the tunnel-barrier height via increasing the channel length enhances the spin injection factor even beyond 0.6 V due to the suppression of the minority-spin current at higher bias. The coexistence of semiconducting behavior, robust intrinsic spin polarization, weak spin–orbit coupling near the Fermi level, and near-perfect spin-injection efficiency over a broad bias range establishes the CrSbSe_3_ nanowire as a promising low-dimensional material for nanoscale spintronic applications, particularly as an efficient spin-filtering tunnel barrier capable of operating under nonequilibrium conditions.

## Supplementary Material



## Notes

Computational methods; Spin-polarized atom and orbital resolved energy band structures of CrSbSe_3_; Highest occupied molecular orbitals for the majority and minority spin channels (PDF)
