# Supplementary material for: CrSbSe3 Nanowire: A Platform for Near-Perfect Spin Selectivity
Source: Nano Lett. 2026 Aug 21;26(38):12734–41. doi: 10.1021/acs.nanolett.6c02702 (PMC13632583; doi:10.1021/acs.nanolett.6c02702)
Supplement: Supplementary file 1 [file nl6c02702_si_001.pdf]

$CrSbSe_3$ Nanowire: A Platform for Near-Perfect Spin Selectivity

Alyssa Horne,[†,¶] Yongmei M. Jin,[‡] and Ranjit Pati[*,†,¶]

†*Department of Physics, Michigan Technological University, Houghton, MI 49931, USA*

‡*Department of Materials Science, Michigan Technological University, Houghton, MI 49931, USA*

¶*Henes Center for Quantum Phenomena, Michigan Technological University, Houghton, MI 49931, USA*

*E-mail: patir@mtu.edu

Computational Methods

Periodic DFT for electronic structure calculation of the $CrSbSe_3$ nanowire:

The computational supercell comprised of 2 Cr, 2 Sb and 6 Se atoms, corresponding to a single unit cell of the $CrSbSe_3$ nanowire, which was periodically repeated in all three spatial directions to model the nanowire structure. To eliminate spurious interactions between the nanowire unit and its periodic images along the y-and z-directions in the supercell, we included a vacuum region of 25 Å along transverse directions. All calculations were carried out using the Vienna Ab initio Simulation Package (VASP) [1, 2] within a plane wave basis set. The kinetic energy cutoff for the plane-wave expansion was set to 700 eV. Electron–core interactions were treated using the projector augmented-wave (PAW) method [3,4] and the exchange–correlation functional was described by the Perdew–Burke–Ernzerhof (PBE) [5] form of the generalized gradient approximation (GGA). Structural optimization was performed until the residual force on each atom were less than 1×10^{-3} eV/Å with a total energy convergence threshold of 1×10^{-8} eV. The Brillouin zone was sampled with a dense 5×1×1 Monkhorst Pack k-point mesh. To mitigate the self-interaction errors inherent to semi-local functionals, we also calculated the electronic band structure using the HSE06 hybrid functional that replaces part of the short-range semi-local PBE exchange with exact Hartree-Fock exchange [6]. In addition, non-collinear spin calculations including spin-orbit coupling (SOC) [7,8] were performed to investigate the SOC-induced band splitting.

Spin-dependent quantum transport of the $CrSbSe_3$ nanowire tunnel junction:

To investigate the spin-dependent quantum transport properties of the $CrSbSe_3$ nanowire tunnel junction, we constructed a prototypical 7×1×1 supercell within a two-probe open-device framework. The left and right terminal cell units were treated as extensions of semi-infinite electrodes that retain the characteristics of the infinite nanowire. The central region, comprising five-unit cells of length 18.75 Å, served as the active scattering channel for transport calculations. Unlike the metallic contacts commonly employed as source and drain electrodes in experimental devices, the channel and the semi-infinite electrodes in our model are composed of the same $CrSbSe_3$ nanowire, thereby forming an ideal, atomically coherent interface with negligible contact resistance and conductivity mismatch. This idealized junction enables the intrinsic spin-dependent transport characteristics of the nanowire channel to be isolated without introducing additional interface effects associated with metal-semiconductor contacts. Furthermore, the spin-polarized electronic states of the $CrSbSe_3$ nanowire provide an intrinsic spin-selective tunneling pathway, suggesting that the material can inherently support efficient spin filtering without requiring an additional tunnel barrier to overcome the conductivity mismatch typically encountered in conventional metal/semiconductor spintronic junctions. In contrast to the k-space approach used to calculate electronic structure and energy band diagram of the infinite nanowire, here, we resorted to a real space, spin-unrestricted, orbital-dependent *a posteriori* DFT formalism to evaluate the bias-dependent spin-polarized Green's function for the two-probe junction.[9–11] The

many electron wavefunction was represented by a single Slater determinant constructed from atom centered Gaussian orbitals [12]; the double-ζ effective core potential basis set (LANL2DZ) was used. This real space approach allowed us to partition the Kohn-Sham-Fock matrix to construct the Green's function and calculate the spin-polarized current within a spin-conserved tunneling framework [11]. An electric dipole interaction term was added to the Hamiltonian during the self-consistent electronic structure calculation of the extended 7×1×1 structure to incorporate the bias effect; both the first- and higher-order Stark effects were explicitly included [11]. During the self-consistent calculation, stringent convergence criteria with ultra-fine integration grid [12] were employed to ensure fully converged electronic structures. The convergence thresholds for the total energy, root mean square electronic density difference and maximum electronic density difference were set at 10^{-8}, 10^{-8}, and 10^{-6} a. u. respectively. The exchange–correlation effects were described using the Becke's three-parameter hybrid functional (B3LYP) [12,13], which incorporates a fraction of exact Hartree–Fock exchange and thereby partially corrects self-interaction errors. This hybrid treatment has been shown to significantly improve the electronic structure description of semiconductor and quantum transport properties in nanoscale junctions, particularly in comparison to semi-local functionals [14]. In addition, the density of states obtained with B3LYP for 3d transition-metal systems has been demonstrated to be in good agreement with results from embedded dynamical mean-field theory [15], supporting its reliability for treating system with transition metals. We considered two additional device structures with different channel lengths of 11.25 Å and 26.25 Å to investigate the role of nanowire channel length on spin-selectivity.

References

[1] Kresse, G.; Furthmüller, J. Efficiency of Ab-Initio Total Energy Calculations for Metals and Semiconductors Using a Plane-Wave Basis Set. Computational Materials Science 1996, 6, 15–50

[2] Kresse, G.; Furthmüller, J. Efficient Iterative Schemes for Ab Initio Total-Energy Calculations Using a Plane-Wave Basis Set. Physical Review B 1996, 54, 11169–11186.

[3] Blöchl, P. E. Projector Augmented-Wave Method. Physical Review B 1994, 50, 17953–17979.

[4] Kresse, I. G.; Joubert, D. From Ultrasoft Pseudopotentials to the Projector Augmented-Wave Method. Physical Review B 1999, 59, 1758–1775.

[5] Perdew, J. P.; Burke, K.; Ernzerhof, M. Generalized Gradient Approximation Made Simple. Physical Review Letters 1996, 77, 3865–3868.

[6] Krukau, A. V.; Vydrov, O. A.; Izmaylov, A. F.; Scuseria, G. E. Influence of the exchange screening parameter on the performance of screened hybrid functionals. The Journal of Chemical Physics 2006, 125, 224106.

[7] Hobbs, D.; Kresse, G.; Hafner, J. Fully unconstrained noncollinear magnetism within the projector augmented-wave method. Physical Review B 2000, 62, 11556.

[8] Steiner, S.; Khmelevskyi, S.; Marsman, M.; Kresse, G. Calculation of the magnetic anisotropy with projected-augmented-wave methodology and the case study of disordered $Fe_{1-x}Co_x$ alloys. Physical Review B 2016, 93, 224425.

[9] Datta, S. Electronic Transport in Mesoscopic Systems; Cambridge University Press, 1995.

[10] Di Ventra, M. Electrical Transport in Nanoscale Systems; Cambridge University Press, 2008.

[11] Mandal, S.; Pati, R. What Determines the Sign Reversal of Magnetoresistance in a Molecular Tunnel Junction? ACS Nano 2012, 6, 3580–3588.

[12] Frisch, M. J. et al. Gaussian~16 Revision C.01. 2016; Gaussian Inc. Wallingford CT.

[13] Becke, A. D. Density-functional thermochemistry. III. The role of exact exchange. The Journal of Chemical Physics 1993, 98, 5648–5652.

[14] Mandal, S.; Pati, R. Mechanism behind the switching of current induced by a gate field in a semiconducting nanowire junction. Physical Review B 2011, 84, 115306.

[15] Mandal, S.; Haule, K.; Rabe, K. M.; Vanderbilt, D. Systematic beyond-DFT study of binary transition metal oxides. npj Computational Materials 2019, 5, 1–8

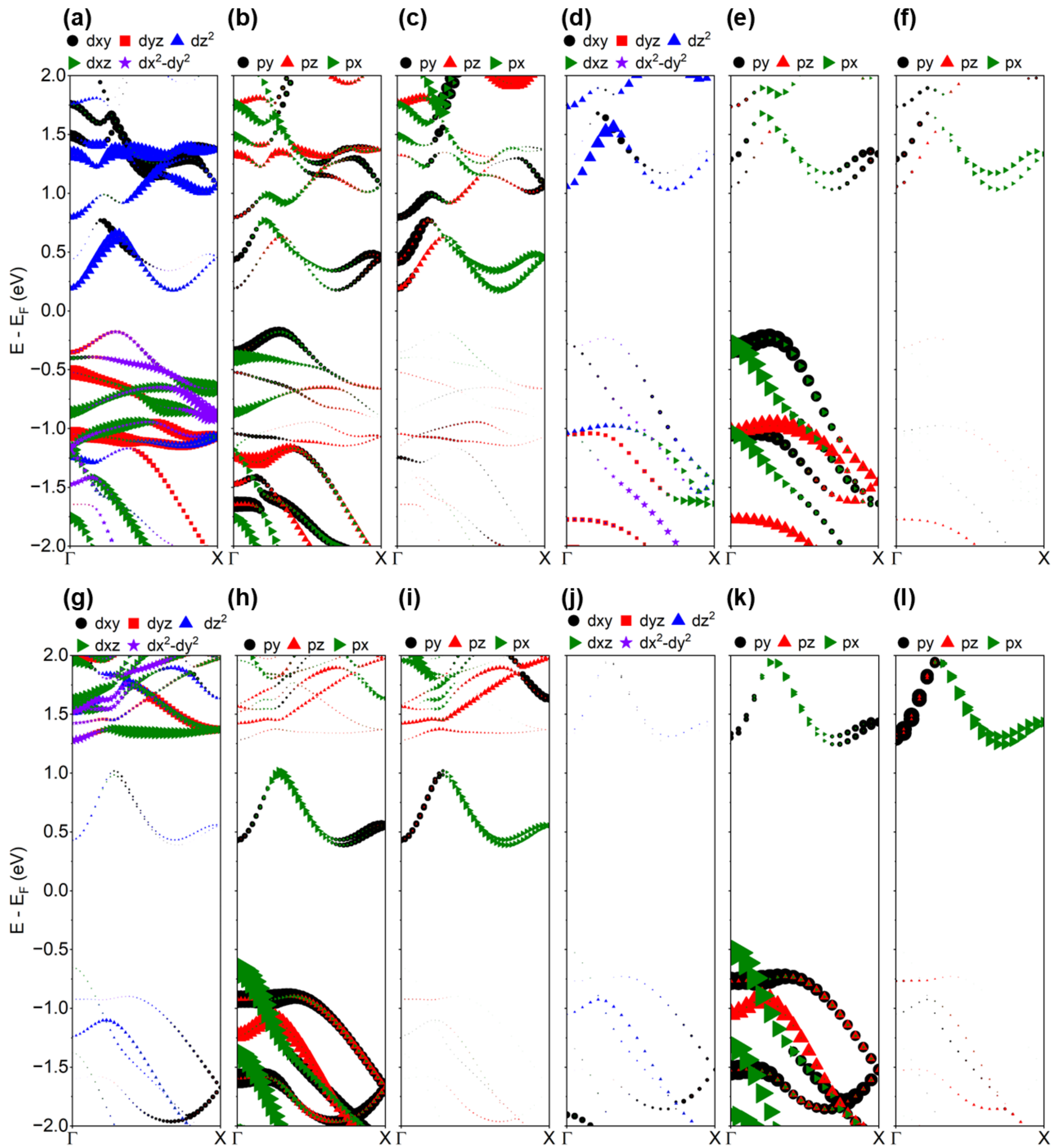

Figure S1. Spin-polarized atom and orbital resolved band structures. The majority spin orbitals from the PBE functional for (a) Cr, (b) Se, and (c) Sb. (d)-(f) are shown likewise for the HSE06 functional. In the same atomic order, the minority spin orbitals for PBE are shown in (g)-(i), and HSE06 in (j)-(l).

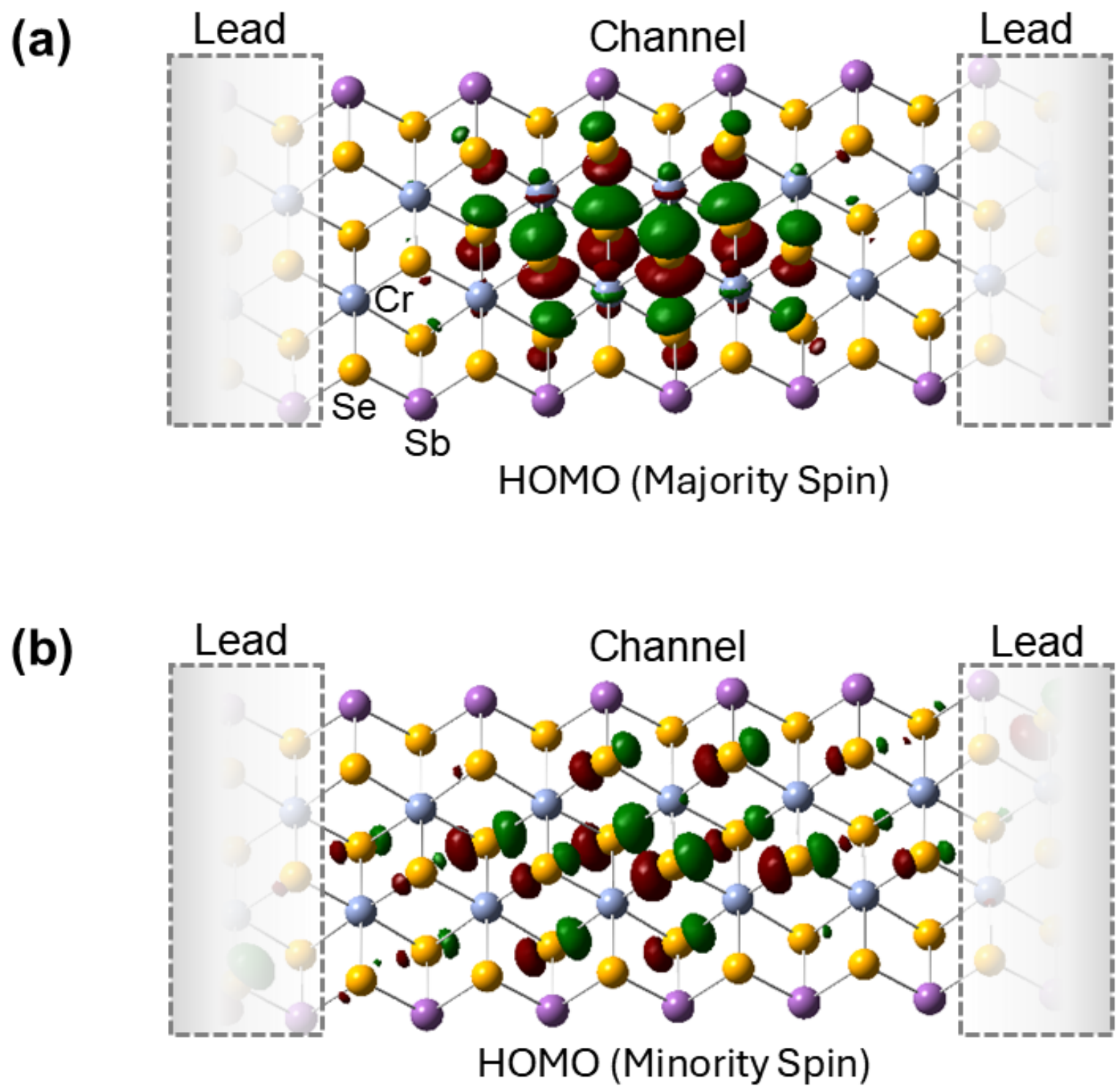

Figure S2. Spin resolved highest occupied molecular orbital (HOMO) for the channel. (a) The HOMO for the majority spin shows a Cr(d)-Se(p) hybridized state, whereas (b) the HOMO for the minority spin is derived mainly from Se p-states.
